# Predicting intensive care unit length of stay: comparing physician assessments with software predictions in a multicenter study

**DOI:** 10.31744/einstein_journal/2025AO1265

**Published:** 2025-11-24

**Authors:** Thiago Tavares dos Santos, Luciana Seidel de Crignis Resende, Leandro Utino Taniguchi, Thiago Gomes Romano, Marcos Soares Tavares, Luciano Cesar Pontes de Azevedo, Fernando Jose da Silva Ramos

**Affiliations:** 1 Hospital Beneficência Portuguesa Mirante Intensive Care Unit São Paulo SP Brazil Intensive Care Unit, Hospital Beneficência Portuguesa Mirante, São Paulo, SP, Brazil.; 2 Universidade de São Paulo Faculdade de Medicina Hospital das Clínicas São Paulo SP Brazil Intensive Care Unit, Hospital das Clínicas, Faculdade de Medicina, Universidade de São Paulo, São Paulo, SP, Brazil.; 3 Hospital Sírio-Libanês Adult Intensive Care Unit São Paulo SP Brazil Adult Intensive Care Unit, Hospital Sírio-Libanês, São Paulo, SP, Brazil.; 4 Hospital São Luiz São Paulo SP Brazil Hospital São Luiz, São Paulo, SP, Brazil.; 5 Hospital Vila Nova Star Intensive Care Unit São Paulo SP Brazil Intensive Care Unit, Hospital Vila Nova Star, São Paulo, SP, Brazil.; 6 Instituto D’Or de Pesquisa e Educação Rio de Janeiro RJ Brazil Instituto D’Or de Pesquisa e Educação, Rio de Janeiro, RJ, Brazil.; 7 Centro Universitário FMABC Santo André SP Brazil Centro Universitário FMABC, Santo André, SP, Brazil.; 8 Hospital Nove de Julho Intensive Care Unit São Paulo SP Brazil Intensive Care Unit, Hospital Nove de Julho, São Paulo, SP, Brazil.; 9 Hospital Israelita Albert Einstein São Paulo SP Brazil Hospital Israelita Albert Einstein, São Paulo, SP, Brazil.; 10 Universidade Federal de São Paulo Discipline of Intensive Care Medicine São Paulo SP Brazil Discipline of Intensive Care Medicine, Universidade Federal de São Paulo, São Paulo, SP, Brazil.

**Keywords:** Length of stay, Prediction algorithm, Quality of health care, Data science, Quality improvement, Databases, factual, Registries, Critical care, Risk factors, Intensive care units

## Abstract

**Objective:**

To compare the predictive abilities of medical professionals and the Epimed Monitor Performance^©^ (EMP) software for intensive care unit length of stay and to assess EMP software effectiveness in identifying patients at risk of prolonged stays.

**Methods:**

This prospective multicenter observational study was conducted in three Brazilian intensive care units, enrolling adult patients admitted between August and December 2019. Data were collected using the Epimed Monitor System^©^. Both physicians and the EMP system predicted intensive care unit length of stay.

**Results:**

A total of 555 patients were included. The median age was 63.3 years (IQR=48.3-74.3), and 58.5% were males. Comorbidities were present in 72%, with 37.2% having cancer. The median intensive care unit length of stay was 3 days (IQR=2-6). Observed length of stay showed a correlation of 0.34 (p<0.001) with physician prediction and 0.36 (p<0.001) with EMP. Categorizing the length of stay improved the predictive performance by 60% for both methods. EMP demonstrated good accuracy in identifying patients at risk of prolonged stay (AUC: 0.76; 95%CI=0.70-0.81).

**Conclusion:**

Absolute prediction of intensive care unit length of stay remains challenging, but period categorization is a viable alternative. EMP aids in identifying patients at risk of prolonged stays and thus can complement clinical judgment. Our findings highlight the value of integrating predictive tools with medical expertise to enhance intensive care unit planning, decision making, and resource allocation.

## In Brief

This prospective multicenter study assessed the accuracy of intensive care unit length of stay predictions made by physicians and the Epimed^®^ Monitor Performance software. A total of 555 intensive care unit admissions across three Brazilian hospitals were analyzed. The correlations between the predictions and the observed length of stay were moderate (r=0.34-0.36). When the length of stay was categorized into periods (<2, 2-5, and >5 days), both approaches achieved approximately 60% accuracy. The Epimed^®^ Monitor Performance demonstrated good performance in identifying patients at risk for prolonged intensive care unit stays (AUC: 0.76). Epimed^®^ Monitor Performance emerged as a useful complementary tool to clinical judgment for intensive care unit decision-making and resource planning.

## Highlights

■ The sample included 555 patients from three hospitals (both public and private), representing diverse care settings.
■ Physician and software predictions showed moderate correlation with observed intensive care unit length of stay, highlighting limitations in absolute prediction accuracy.
■ Categorizing length of stay into time periods (<2, 2-5, >5 days) improved prediction accuracy to approximately 60% for both physicians and Epimed Monitor Performance^®^.
■ Epimed Monitor Performance^®^ identified patients at risk of prolonged intensive care unit stay with an AUC of 0.76, sensitivity of 66.6%, and specificity of 74.2%.
■ Epimed Monitor Performance^®^ can complement clinical judgment, supporting efficient intensive care unit planning and management.

## Graphical abstract

**Figure:**
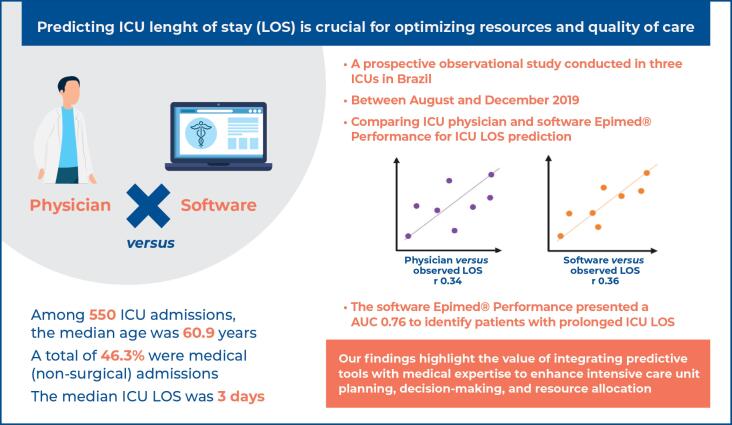

## INTRODUCTION

The increasing demand for intensive care for patients with severe conditions limits intensive care unit (ICU) capacity. This can lead to a shortage of available beds and excessive workload for multidisciplinary teams, ultimately resulting in higher morbidity and mortality rates.^(1,2)^ Currently, ICUs are under strong pressure to improve efficiency and reduce operating cost.^(3,4)^

Since resource use during ICU stay is related to the length of stay (LOS), a shorter stay generally results in lower resource use and cost.^(3)^ Accurate prediction of LOS is essential for optimizing ICU operations and reducing unnecessary cost.^(2,5)^ Furthermore, predicting ICU LOS allows for 1) planning the number of beds and staff needed to meet the demand of a given hospital; 2) identifying patients or groups of patients with longer-than-predicted stays, enabling direct quality improvement; and 3) comparison of average LOS between ICUs (benchmarking).^(4,6)^

International healthcare accreditation organizations, such as the Joint Commission International, require LOS prediction during patient admission and stay.^(7)^ However, LOS predictions by ICU physicians often yield inaccurate results.^(8,9)^

In recent years, several models have been published to predict ICU LOS. However, their clinical utility remains unclear, and there is no consensus on the best method.^(4,10)^ Accurately and precisely predicting ICU LOS is challenging for three main reasons: 1) statistical methods often assume a Gaussian distribution; however, ICU LOS is generally right-skewed; 2) ICU patients form a heterogeneous group with a wide range of complex health problems, each of which may have a different association with LOS; and 3) the relationship between disease severity and LOS varies between survivors and non-survivors. Patients with more severe diseases often stay longer, while those with extremely critical conditions have a higher risk of death, which can lower the average LOS.^(4,11-13)^

Advances in machine learning and artificial intelligence have shown promise in improving the predictability of ICU outcomes, including hospital stays and readmission rates. Despite the challenges of model generalization and data variability, these technologies support better decision-making in clinical care and resource management.^(4,12,14)^ Efforts to develop predictive models and clinical decision-support tools aim to enhance ICU efficiency, reduce avoidable readmissions, and support personalized patient care. More recently, the Epimed Monitor system^©(15)^ a cloud-based platform used for ICU performance management using data from critically ill patients since 2009, has been updated to include the Epimed Monitor Performance^©^ (EMP) software.^(15,16)^ Using each diagnostic category and demographic information, the EMP software estimates the median LOS for each patient at different stages. It also provides an individualized risk of a long stay, defined as greater than the 90^th^ percentile of the LOS for each patient, based on their main diagnosis and clinical conditions.^(15)^

## OBJECTIVE

To compare the potential for estimating observed length of stay between physicians and Epimed Monitor Performance^©^, and to assess the accuracy of Epimed Monitor Performance^©^ in identifying patients at risk for long stays according to the software definition.

## METHODS

### Design and patients

This was a prospective multicenter observational cohort study. The study was conducted in three ICUs of the following hospitals located in São Paulo, Brazil: *Hospital Beneficência Portuguesa de São Paulo*-*BP Mirante* (20 beds), *Hospital Nove de Julho*-Oncology ICU (10 beds), and the emergency ICU of the *Hospital das Clínicas* at the *Faculdade de Medicina, Universidade Federal de São Paulo* (HCFMUSP) (9 beds). None of the participating ICUs had explicit admission and discharge criteria. However, they all followed the guidelines outlined by the Brazilian Federal Council of Medicine (CFM Resolution No. 2156/2016).^(17)^ ICU discharge was made by the ICU physician together with the specialist physician responsible for the patient. Only one hospital in this study (*Hospital BP Mirante*) had a semi-intensive care unit, with 10 beds. All participating ICUs had physicians on duty 24 hours a day, 7 days a week; intensivist-certified daily physicians; and a specialized intensive care multidisciplinary team.

Patients admitted to the participating ICUs between August and December 2019 were consecutively included in the study, and their ICU LOS was predicted by the attending physician. The inclusion criteria were patients aged >18 years who were discharged from the ICU after a stay of >12 h. Patients who had already been admitted to the ICUs before the start of the study were excluded.

Patient demographic information was retrieved from the Epimed Monitor^©^ (Epimed Monitor, Rio de Janeiro, Brazil). The Epimed Monitor^©^ contains structured data prospectively collected from all ICU admissions of the participating ICUs, with a comprehensive characterization of patients regarding demographics, admission diagnosis, comorbidities, disease severity scores, organ support therapies, and ICU and hospital outcomes. The following data were routinely collected by the participating ICUs: age, sex, Charlson Comorbidity Index, diagnosis, SAPS3 score (Simplified Acute Physiology Score), ICU and hospital outcomes, date and time of ICU discharge, destination after ICU discharge, and date and time of hospital discharge.

The Epimed Monitor^©^ also contains the EMP software, which evaluates, based on clinical information from the first day of admission, the predicted median ICU LOS and the risk of long stay, for each patient in the ICU. The results are expressed as the median and risk of long stay, considered as the risk of staying above the 90th percentile of LOS for each patient based on their main diagnosis and clinical conditions.^(15)^

Admitting physicians were asked to collect data on the predicted ICU LOS of each patient admitted during the study period. In one hospital, a printed form was used for this, and in the other two hospitals, the data were retrieved from the patients’ electronic medical record. Data related to admitting physicians (age, sex, years since medical school graduation, years of experience in intensive care medicine, and certification in intensive care medicine) were also collected.

The study was approved by the Research Ethics Committee of *Hospital Beneficência Portuguesa de São Paulo* (coordinating center), under CAAE: 03190618.3.1001.5483: # 3.082.238 and by the ethics committees of the other participating centers. This study followed the STROBE guidelines for observational studies.^(18)^

### Statistical analysis

Convenience sampling was used in this study. Continuous variables are presented as medians and interquartile ranges and compared using the Mann-Whitney U test due to non-normal distribution according to the Kolmogorov-Smirnov test. Categorical variables are presented as absolute values and percentages and compared using the chi-squared test or Fisher's exact test, as appropriate.

In this study, the ICU LOS refers to the period between ICU admission and ICU discharge. The observed LOS was used as the reference for analyses. Thus, the observed LOS was compared with the ICU LOS predictions by physicians and EMP. A multivariate linear regression model with an intercept was used to study the behavior of the predictions. The estimates (coefficients), standard errors, t-values, p-values, root mean square error (RMSE), mean absolute error (MAE), and R-squared (R^2^) values were reported. The intercept represents the expected value of the dependent variable when it is zero. The R^2^ values indicate the proportion of variance in the dependent variable that can be explained by the independent variable.

The intraclass correlation coefficient (ICC) was used to assess the reliability and consistency of the predictions. This allowed the comparison of different ICC values, confidence intervals, and significance levels. ICC values, along with lower and upper confidence intervals, and p-values were used to evaluate the consistency and reproducibility of the measures.

Additionally, we stratified the LOS into three periods: <2 days, 2-5 days, and >5 days, based on a previous study.^(9)^ When the ICU LOS was longer than that predicted by the physician or EMP, the prediction was labeled as underestimated. When the ICU LOS was shorter than that by the physician or EMP, the prediction was labeled as overestimated. Cohen's Kappa test was used to measure the level of agreement between the predictions made by physicians and EMP in terms of the observed LOS for each group. The strength of agreement was described according to the Kappa statistic as follows: ≤0.2 poor, 0.21-0.4 fair, 0.41-0.6 moderate, 0.61-0.8 good, and 0.81-1.0 very good.^(19)^ Correlation analyses were performed to examine the relationship between the following pairs of variables: observed LOS and physician predictions, observed LOS and EMP predictions, and physician and EMP predictions.

The accuracy of the EMP software in identifying patients at risk for prolonged ICU stay was assessed using a receiver operating characteristic (ROC) curve. In this analysis, a prolonged ICU stay was defined as any period exceeding the 90th percentile of the median predicted LOS for a specific disease according to the database provided by the EMP software, considering the specific patient and diagnosis.

p<0.05 was considered significant. Data were analyzed using R software (version 4.3.2).

## RESULTS

**Figure 1:**
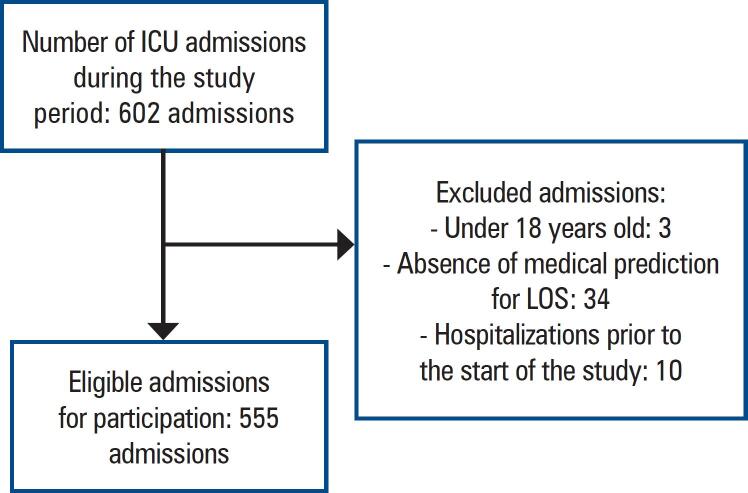
Patient flow and study inclusion

ICU: intensive care unit; LOS: length of stay.

Based on the inclusion criteria, 555 patients were included. Among them, 70.1% (397/555) were admitted to the ICU of *Hospital BP Mirante* and 22.3% (124/555) to the Emergency ICU of HCFMUSP (Figure 1). Patient profiles are detailed in table 1. The median age was 63.3 (48.3-74.3) years, and 58.5% (325/555) were males. Comorbidities were present in 72% (399/555) of the patients, with 37.2% (207/555) having cancer. Among the admissions, 46.3% (257/555) were for clinical reasons, 41.1% (228/555) for elective surgery reasons, and 12.6% (70/555) for emergency surgery reasons. The main source of patients was the surgical center (50.9%, 283/555). The most common clinical diagnosis leading to ICU admission was infection or sepsis (38.1%, 98/257), followed by neurological causes (20.2%, 52/257). Among surgical patients, the most common classifications were neurosurgery (18.8%, 56/298) and cardiac surgery (14.7%, 44/298). The average SAPS3 score of the patients was 45.8±15.2, and the median ICU LOS was 3 (2-6) days. The most common discharge destinations were inpatient units (56.7%, 283/555) and semi-intensive care units (34.7%, 173/555). Regarding outcomes, 6.6% (37/555) of the patients died in the ICU and 11.7% (65/555) died in the hospital. Palliative care decisions were made in 6% (34/555) of the patients.

**Table 1:** Characteristics of the patients included in the study

Patient characteristics		
Sex, n (%)		
	Male	325 (58.5)
Age		
	Mean±SD	60.9±17.8
	Median	63.3 (48.3-74.3)
Comorbidity, n (%)		
	Yes	399 (71.8)
	Heart failure (NYHA Class II-IV)	61 (11)
	Chronic kidney disease (on dialysis)	33 (5.9)
	Cirrhosis (Child A-C)	11 (2)
	COPD	18 (3.2)
Cancer, n (%)		
	Solid tumor	193 (34.7)
	Hematologic	14 (2.5)
	Staging locoregional	103 (18.5)
	Staging metastatic	90 (16.2)
	Diabetes	142 (25.5)
Type of admission, n (%)		
	Emergency surgery	70 (12.6)
	Elective surgery	228 (41)
	Medical	258 (46.4)
Source, n (%)		
	Operating room	283 (51)
	Emergency room	162 (29.2)
	Ward	60 (10.8)
	Other ICU in the same hospital	7 (1.2)
	Semi-intensive care unit	12 (2.1)
	Transfer from another hospital	24 (4.3)
	Others	7 (1.4)
Surgical conditions		
	Neurosurgery	56 (18.8)
	Cardiac surgery	44 (14.8)
	Abdominal/Retroperitoneal surgery	30 (13.4)
	Colon/Sigmoid/Rectal surgery	22 (7.38)
Clinical conditions, n (%)		
	Infection/Sepsis	98 (38.1)
	Neurological	52 (20.3)
	Cardiovascular	32 (12.4)
	Gastrointestinal	24 (9.3)
SAPS3 score		
	Mean±standard deviation	45.8±15.2
	Median	44 (34-57)
Length of Stay		
	Mean±standard deviation	5.6±8.6
	Median	3 (2-6)
Discharge from ICU, n (%)		
	Hospital ward	283 (56.7)
	Semi-intensive care unit	173 (34.7)
	Other ICUs	14 (2.8)
	Transfer to another hospital	8 (1.6)
	Other	21 (4.2)
Outcome, n (%)		
	ICU mortality	37 (6.7)
	Hospital mortality	65 (11.7)

SD: standard deviation; NYHA: New York Heart Association; COPD: Chronic obstructive pulmonary disease; ICU: intensive care unit.

### Physicians’ characteristics

Data from 65 physicians who predicted ICU LOS for the patients were evaluated, with 61.5% (40/65) being males, with an average age of 38.7±5.7 years. The median number of years since graduation was 12 (9-20) years, and the median number of years of ICU experience was 10 (7-18) years. Among the physicians, 38.5% (25/65) were attending intensivists and 67.6% (44/65) were certified in intensive care medicine. Attending physicians predicted ICU LOS in 60.0% (332/555) of the cases.

### Intensive care unit length of stay prediction

**Figure 2:**
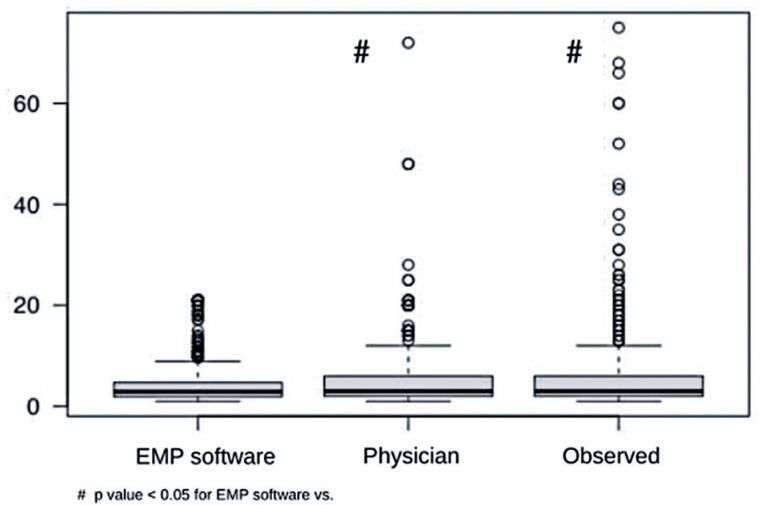
Comparison of intensive care unit stay predictions

The median observed ICU LOS was 3 (2-6) days. Physician prediction was 3 (2-6) days, and the EMP prediction was 2.9 (2-4.7) days (p<0.05) (Figure 2).

The Pearson correlation coefficient between the EMP prediction and observed ICU LOS was 0.36 (p<0.001), indicating a moderate positive linear relationship, while that between the physician prediction and observed ICU LOS was 0.34 (p<0.001); between physician prediction and EMP prediction, the Pearson correlation coefficient was 0.46 (p<0.001), also indicating a moderate positive linear relationship.

The intercept model for ICU LOS prediction by EMP started with an intercept of 2.42 days (p<0.001). The regression coefficient of LOS prediction by the software was 0.69 (p<0.001). The coefficient of determination of the model was 0.13, indicating a modest explanatory power (Table 2).

**Table 2:** Intraclass correlation coefficient with intercept

Variable	Estimate	Standard deviation	T-value	p value	RMSE	MAE	R^2^
Intercept	2.42	0.48	5.02	<0.01	8.06	4.17	0.13
EMP	0.69	0.07	9.35	<0.01			
Intercept	2.85	0.46	6.14	<0.01	8.13	4.11	0.12
Medical team	0.49	0.05	8.72	<0.01			

Moreover, the intercept model for ICU LOS prediction by physicians started with an intercept of 2.85 days (p<0.001). The coefficient for physician prediction was 0.49 (p<0.001), and the coefficient of determination of the model was 0.12, showing modest explanatory power (Table 2). For the comparative analysis between the physician and EMP prediction, the intercept of the model was 2.66 days (p<0.001). The regression coefficient for the prediction by the software in this comparison was 0.61 (p<0.001). The model had a coefficient of determination of 0.21, suggesting a slightly higher explanatory power than that of the two models described above (Table 2).

When the ICU LOS predictions were categorized into periods, most EMP predictions were classified as accurate (60.5%), while others were classified as underestimation (23.9%) and overestimation (15.5%). Both physician and EMP predictions had an accuracy of approximately 60%.

Table 3 presents the percentage values of the EMP predictions for the categorized periods. The Cohen's Kappa test value was 0.51 (p<0.001), indicating moderate agreement between the EMP estimate and observed ICU LOS. Data related to physician predictions during the categorized periods are presented in table 4. The Cohen's Kappa test value was 0.48 (p<0.001), indicating a moderate level of agreement between the predicted and observed LOS.

**Table 3:** Agreement between Epimed Monitor Performance^©^ prediction and observed intensive care unit length of stay

	Observed ICU LOS						
	<2 days		2–5 days		>5 days		Total
	Total	%	Total	%	Total	%	
<2 days	76	53.5	58	40.8	8	5.6	142
2-5 days	56	17.9	189	60.6	67	21.5	312
>5 days	6	5.9	24	23.8	71	70.3	101
Total	138		271		146		555

ICU: intensive care unit; LOS: length of stay.

**Table 4:** Agreement between physician prediction and observed intensive care unit length of stay

	Observed ICU LOS						
	<2 days		2-5 days		>5 days		Total
	Total	%	Total	%	Total	%	
<2 days	26	63.4	14	34.1	1	2.4	41
2-5 days	105	28.2	217	58.3	50	13.4	372
>5 days	7	4.9	40	28.2	95	66.9	142
Total	138		271		146		555

ICU: intensive care unit; LOS: length of stay.

**Figure 3:**
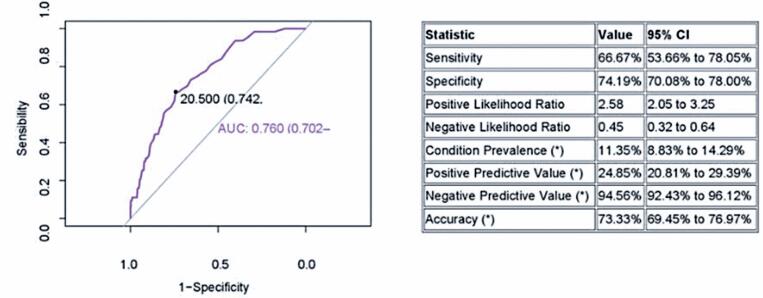
Accuracy of the Epimed Monitor Performance^©^ software in predictiong patients with prolonged hospitalization

When analyzing the ability of EMP to identify patients at risk for prolonged ICU stay, we found an AUC of 0.76 (0.70-0.81), an accuracy of 73.3% (69.4%-76.9%), sensitivity of 66.6% (53.6%-78.1%), and specificity of 74.2% (70.1%-78.0%). Figure 3 shows the ROC curve and results.

## DISCUSSION

Our study suggests that both physicians and EMP had moderate accuracy in predicting ICU LOS at the time of admission. However, approximately 60% of LOS predictions in the ICU were considered accurate when the LOS was categorized into periods.

Proper planning of ICU occupancy is essential for adequate resource management and planning of elective or emergency ICU admissions.^(6)^ Issues of high occupancy rates and lack of beds for critically ill patients are frequent and have become even more evident during the COVID-19 pandemic.^(20,21)^ LOS is used as a quality indicator and is one of the main determinants of resource use, cost, and ICU efficiency.^(6,22)^ Adequate prediction of ICU LOS, along with knowledge of other indicators, such as occupancy rate, average LOS, and bed turnover, can help develop measures to improve ICU bed flow, enhance communication between ICU teams and families, and allow a more efficient expectation management. ^(4)^

Previous studies have shown that physicians make inaccurate ICU LOS predictions, regardless of patient profile and intensivist experience.^(8,9)^ Our results are consistent with these previous studies, as the physicians herein showed a modest ability to predict LOS in absolute terms, but when the LOS was categorized into periods, they were able to accurately predict LOS in 60% of admissions.

In recent decades, technological advances and new statistical methods have made it possible to develop new predictive models for estimating ICU LOS.^(4,10-14,20,23)^ However, a systematic review showed that among the studies analyzed, no model fully met the requirements both for general patients and subgroups of patients with an expected prolonged ICU stay.^(10,13,14)^ The main parameters assessed by these models include patient severity scores, source of admission, age, mechanical ventilation, Glasgow Coma Scale score on admission, comorbidities, hospital LOS before ICU admission, organizational factors, and other predictors, and the type of statistical model used for prediction affects results. Most studies used multivariate linear regression and did not test other approaches to compare accuracy. In contrast, Peres et al. suggested that, in addition to commonly used statistical methods, such as the generalized linear model, linear mixed model, and ordinary least squares, a data-driven approach, such as support vector regression, which shows superior results, should be used.^(4)^ Furthermore, the authors recommended that results should be standardized and expressed in terms of prediction errors using RMSE and MAE, which can help researchers evaluate the best models.^(4)^ In the present study, physician predictions showed an RMSE of 8.13, MAE of 4.11, and R^2^ of 0.12, while EMP predictions exhibited an RMSE of 8.06, MAE of 4.17, and R^2^ of 0.12. These results demonstrate the limitations of both physicians and EMP in predicting ICU LOS in absolute terms. However, these results are similar to those of other studies.^(14)^ In a cohort of 32,667 patients in 83 ICUs, Verburg et al. evaluated ICU LOS and reported RMSE of 7.28, MAE of 3.43, and R^2^ of 0.15.^(14)^

Early identification of patients at a high risk of prolonged stay poses an additional challenge, as this group of patients incurs the highest costs to the healthcare system and stands to benefit the most from specific therapies, improved communication, and strategies for managing expectations. While a small percentage of ICU patients have a prolonged LOS (4-11%), this population is proportionally responsible for up to 52% of ICU days.^(24)^ Recently, Peres et al. developed a data-driven model using data from a cohort of 99,000 patients in 93 Brazilian ICUs. The developed model showed an RMSE of 3.82, MAE of 2.52, R^2^ of 0.36, and AUC of 0.87 for identifying patients at risk of prolonged stay, defined as >14 days.^(23)^ Herein, we used the criterion defined by the EMP software to identify patients at risk of prolonged stay and found an AUC of 0.76 (0.70-0.81). There is no consistent definition in the literature regarding what constitutes prolonged ICU stay. The literature presents different definitions, such as 7, 14, or 21 days, regardless of the diagnosis and unit profile.^(24-26)^ This universal definition has been criticized because the main diagnosis significantly influences what constitutes a prolonged LOS. Thus, for patients undergoing elective surgeries with a low complication risk, the cutoff value for prolonged LOS should be lower than that for a patient admitted, for example, for severe community-acquired pneumonia and on mechanical ventilation. In this regard, the EMP software has the advantage of considering and adjusting the definition of LOS according to the main diagnosis and clinical condition of patients.

While our results showed that both physicians and EMP have a modest ability to predict LOS in absolute terms, when we evaluated prediction by period of stay, we found that approximately 60% of the time, both physicians and EMP accurately predicted the LOS. These data are superior to those of a single-center Brazilian study in which only 50% of the predictions were correct.^(9)^

The strengths of this study include the representativeness of the sample (from different hospitals: private and public) although being small, and the evaluation of not only the absolute prediction capability of physicians and EMP but also period-based predictive abilities, which can be used for bed planning and the adoption of measures to improve ICU performance. Another point to consider is that this is the first study to evaluate EMP in clinical practice.

Despite these strengths, this study has some limitations that must be considered when interpreting the results. Although data were collected from three ICUs – two private and one public university hospital – all the institutions were located in the city of São Paulo. Therefore, the sample may not be representative of ICUs in other regions or of those with different care profiles. Further, the sample was obtained through convenience sampling over a relatively short period of three months, which limits the external validity of the findings.

Additionally, the study did not include subgroup-specific validations (*e.g*., comparisons between public and private institutions, different admission types, or clinical profiles), limiting the assessment of model performance across different care settings. Despite the involvement of hospitals with varying organizational structures and patient populations, the absence of stratified analyses and external validation reduces the generalizability of the results.

Furthermore, the organizational characteristics of the participating ICUs may have influenced both the LOS and predictive performance. For example, only one center (*Hospital BP Mirante*) had an intermediate care unit, potentially facilitating earlier ICU discharge and affecting the LOS distribution. However, no adjustments were made for these institutional factors, which may have affected both the observed outcomes and predictive accuracy of the models.

In addition, in terms of predictive accuracy, both physicians and EMP demonstrated limited performance for absolute LOS prediction, highlighting their limited ability to explain the observed variability in LOS, contrary to previous interpretations suggesting modest performance. Revising this interpretation aligns with the technical definition of the coefficient of determination and promotes a more accurate understanding of the model performance.

To improve prediction, we categorized LOS into three periods: <2 days, 2-5 days, and >5 days, based on the study by Nassar et al.,^(9)^ which are clinically meaningful for discharge planning and resource allocation. Although this categorization improved the prediction accuracy, it also introduced limitations. In particular, our sample exhibited a skewed LOS distribution favoring shorter stays, which may have biased model calibration and affected the categorization accuracy.

Finally, it is important to note that EMP uses a large, real-time updated database. As the software evolves and incorporates more recent data, its performance may vary over time.

## CONCLUSION

In this multicenter study, we found that both physicians and Epimed Monitor Performance^©^ demonstrated limited ability to accurately predict intensive care unit length of stay in absolute terms, with no significant differences between the two approaches. However, the predictive accuracy improved when length of stay was categorized into clinically relevant periods (<2, 2-5, and >5 days), suggesting that this strategy may serve as a practical alternative to absolute predictions. Despite limitations, such as loss of granularity and imbalance among categories, Epimed Monitor Performance^©^ showed acceptable performance in identifying patients at high risk for prolonged intensive care unit stays. These findings support the use of Epimed Monitor Performance^©^ as a complementary tool to clinical judgment. Overall, our results highlight the value of integrating predictive models with clinical expertise to enhance decision making, planning, and resource management in intensive care settings. Future studies should explore whether combining clinical predictors with machine learning-based approaches can further improve the accuracy of intensive care unit length of stay predictions.
